# Buried Penis: Evaluation of Outcomes in Children and Adults, Modification of a Unified Treatment Algorithm, and Review of the Literature

**DOI:** 10.1155/2013/109349

**Published:** 2013-12-29

**Authors:** I. C. C. King, A. Tahir, C. Ramanathan, H. Siddiqui

**Affiliations:** Department of Plastic and Reconstructive Surgery, James Cook University Hospital, Middlesbrough TS4 3BW, UK

## Abstract

*Introduction*. Buried penis is a difficult condition to manage in children and adults and conveys significant physical and psychological morbidity. Surgery is often declined due to morbid obesity, forcing patients to live in disharmony for years until the desired weight reduction is achieved. No single operative technique fits all. We present our experience and surgical approach resulting in an improved algorithm unifying the treatment of adults and children. *Methods*. We conducted a retrospective analysis of patients treated for buried penis between 2011 and 2012. All patients underwent penile degloving and basal anchoring. Penile shaft coverage was achieved with skin grafts. Suprapubic lipectomies were performed on adult patients. *Results*. Nine patients were identified: four children and five obese adults. Average postoperative stay was three days for children and five for adults. Three adults were readmitted with superficial wound problems. One child had minor skin breakdown. All patients were pleased with their outcomes. *Conclusion.* Buried penis is a complex condition, and treatment should be offered by services able to deal with all aspects of reconstruction. Obesity in itself should not delay surgical intervention. Local and regional awareness is essential to manage expectations in these challenging patients aspiring to both aesthetic and functional outcomes.

## 1. Introduction

The buried penis is widely regarded as a condition which is difficult to manage both in children and in adults. Buried penis was first described by Keyes in 1919 as follows: “absence of the penis exists when the penis, lacking its proper sheath of skin, lies buried beneath the integument of the abdomen, thigh or scrotum” [1]. Buried penis has most frequently been discussed in relation to the paediatric population [2–8], with congenital and iatrogenic aetiologies identified. Buried penis in adults may have a congenital component in some cases but is largely regarded as being an acquired condition as a consequence of obesity, lymphoedema, penile trauma (including circumcision), and persistent infection, usually in the presence of diabetes.

In children, presentation is often driven by parental concerns over urinary symptoms and penile size. Adult patients present with symptoms which have a profound impact on their lives. Patients can complain of being unable to pass urine while standing—and sometimes sitting—without soiling themselves, of having recurrent penile and urinary infections which are uncomfortable and antisocial, or being unable to achieve erections without pain, or to accomplish successful vaginal penetration with the consequences of damaged relationships and lowered self-esteem. Prompt recognition and treatment of these symptoms in both adults and children are thus essential to reconstruct more normal appearance and function.

**Figure 1:**
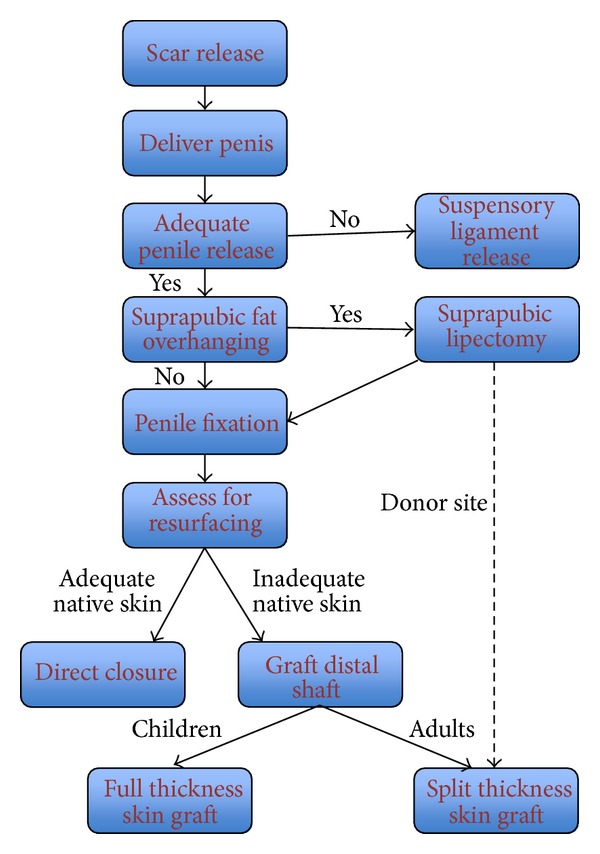
Treatment algorithm for adults and children with buried penis (adapted from [9]).

The complex interaction of significant physical and psychological symptoms of patients with a buried penis means that treatment must be tailored to the individual. Indeed, within the literature, no single operative technique has been described to meet all patients' needs. Algorithms have been advocated for treatment of adults with buried penis [9, 10] to take into account the different surgical approaches to this problem. We present our experience of buried penis treatment in adults and children, using a single surgical technique which incorporates an understanding of the aetiology of buried penis in the two populations (Figure 1), unifying management, and streamlining our practice into a modified treatment algorithm.

## 2. Methods

A retrospective analysis was performed for all patients who had undergone treatment for a buried penis in James Cook University Hospital between 2011 and 2012. All patients had been treated under a single surgeon.

**Figure 2:**
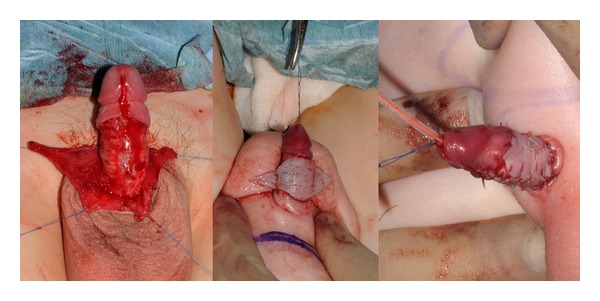
Penile skin coverage demonstrating delivery of the penis from tethering tissue and resurfacing with fenestrated skin graft draped dorsally to recreate the ventral raphe.

Under general anaesthetic with antibiotic cover (Co-amoxiclav), the penis is delivered by degloving the surrounding tissues. A urethral catheter can be inserted to enable control of the penis and some degree of protection of the ventral urethra during dissection, if required. A 4-0 nylon stitch is placed through the glans to give further control and enable traction of the penis. The penile shaft is circumferentially degloved from a distal coronal incision, leaving 1 cm of subcoronal cuff, to the penile base along the subdartos plane allowing for any chordee encountered to be released and to preserve the dorsal neurovascular bundle (Figure 2). Infected or scarred tissue is removed as necessary and sent for laboratory analysis. The penopubic and penoscrotal angles are reconstructed using 3-0 PDS sutures between the tunica albuginea and dartos fascia and dermis at the penile base, placed in the 12, 7, and 4 o'clock positions.

**Figure 3:**
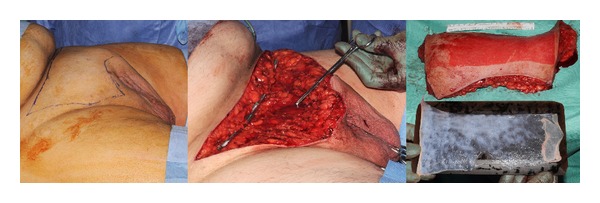
Suprapubic lipectomy can uncover the penile base position (being pointed centrally) and provide a useful skin graft donor site.

In adults, who are all obese in our population, the procedure incorporates a suprapubic lipectomy. Marked preoperatively, the patients have suprapubic lipectomy through a “W” shaped incision based 2-3 cm cranial to the penile base (Figure 3). If skin is required for shaft and/or glans resurfacing, the skin is harvested from this region using a dermatome (setting 12) as a sheet graft. The excess tissue is weighed, and the wound is closed with Scarpa's fascia and two-layer skin sutures with PDS and monocryl. One or two suction drains are inserted and secured with silk.

Penile skin is redraped as necessary with native skin, with priority given to the proximal end of the penis as this will facilitate penile fixation. Skin is joined on the ventral surface to mimic the ventral raphe. Where skin has been removed or native skin is insufficient, penile coverage is completed using either full thickness skin grafts harvested preferentially from the groin in children, or split skin graft from either the excised suprapubic skin or from the thigh in adults (Figure 3). Grafts are held in place with 5-0 vicryl rapide circumferential and quilting sutures (Figure 2). The distal coronal incision is closed circumferentially with an interrupted 5-0 vicryl rapide suture. Penile dressing is achieved using a nonadherent vaseline-impregnated Jelonet dressings covered with a proflavine-soaked gauze support dressing. The abdomen is dressed with steristrips and an adherent dressing. A course of antibiotics is prescribed for a week, and wounds are reviewed on the third postoperative day with discharge home if mobilizing well, then coming back for graft check and catheter removal after a week. Patients are followed up as outpatients within six weeks, at six months, and remain under review for at least a further year.

## 3. Results

A total of nine patients were treated for buried penis between 2011 and 2012 (Table 1). Five patients were adult men with an average age of 51 years (range 28–76). The five adults had an average BMI of 45. Presentation by the adult group consisted of a range of symptoms which were in all cases multifactorial and included difficulty passing urine (*n* = 3), and recurrent urinary infections (*n* = 1), sexual dysfunction, including pain on erection and impossible penetration (*n* = 3), aesthetic concerns (*n* = 3), and recurrent infections of the penis itself, including recurrent phimosis and lichen sclerosis et atrophicus (balanitis xerotica obliterans, BXO) (*n* = 4) and Fournier's gangrene (*n* = 1). Four patients had undergone previous circumcisions, and the same patients were diabetic but nonsmokers.

The remaining four patients were children with an average age of 6 years (range 8 months–12 years). None were obese or had undergone previous penile surgery; indeed otherwise they were fit and well and developmentally normal. All four presented with poorly controlled urinary streams, and parents were uniformly concerned about the size of their child's penis. Comorbidities included hypogonadism (*n* = 1), glandular hypospadias (*n* = 1) and phimosis (*n* = 1).

All patients had penile degloving and penile fixation, and all but the youngest child required skin grafts for coverage of the penile shaft. Four of the adults underwent suprapubic lipectomy with an average of approximately one kilogram of tissue removed. Additional adult procedures during the operation included a partial glansectomy (*n* = 1) following recurrent BXO and suspensory ligament release for another to achieve a functional shaft length. Additional intraoperative procedures for the children included a frenuloplasty (*n* = 1), a single-stage Snodgrass hypospadias repair (*n* = 1), and a megaprepucectomy (*n* = 1).

Operative duration without lipectomy was 2.6 hours on average, whereas the average operation for those having lipectomy was 3.8 hours. Children remained in hospital for 3 days on average and adults remained for 5.5 days. The patient with Fournier's gangrene had a longer hospital stay (14 days) due to his acute illness. Three adults were readmitted: two due to poor bodily hygiene resulting in superficial wound infections and the third who experienced some wound dehiscence when exerting himself. The child who did not undergo skin grafting had some ventral shaft skin loss which healed by secondary intention (Table 2).

**Figure 4:**
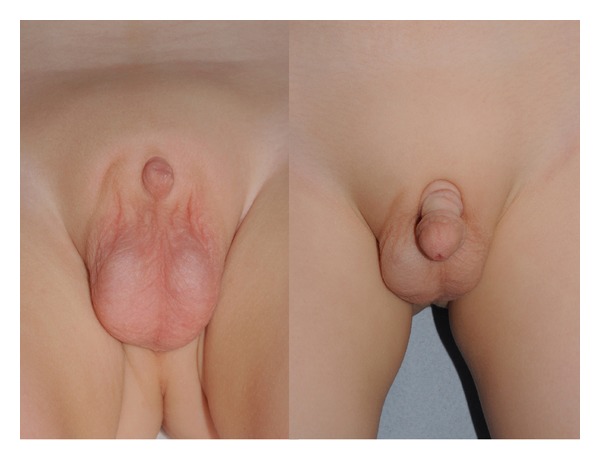
Buried penis in a 2-year-old child and the postoperative skin grafted penis at the age of 4.

**Figure 5:**
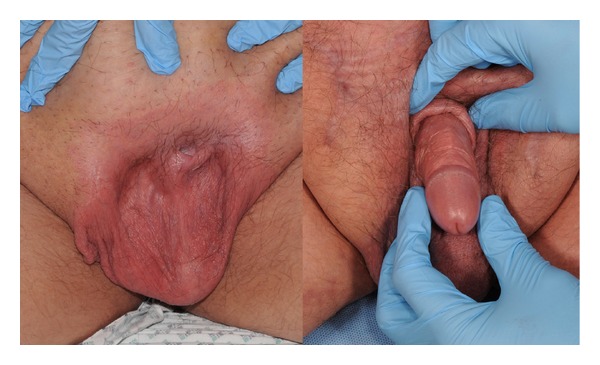
An adult with buried penis who underwent penile shaft resurfacing.

All patients were followed up, ranging from 6 to 30 months; the shorter followup is due to patient choice following poor compliance (Table 3). All patients reported much improved urinary function, particularly with regard to standing micturition which all felt able to accomplish following the surgery. Sensation over the grafts significantly varied. None reported urinary tract infections or recurrence of BXO. The teenagers and adults reported painless, effective erections, and the few who were sexually active were able to achieve painless, effective vaginal penetration (Figures 4 and 5). No buried penis recurred, and all patients stated that they were pleased or very happy with their outcome.

## 4. Discussion

Clarity in the approach to buried penis management is hindered by the confusing use of interchangeable terminology to describe the condition. A penis may be referred to as buried [1], webbed [11], concealed [12], inconspicuous [13], or entrapped [14]. Micropenis is an entirely distinct condition with separate aetiological and anatomical features and care must be taken to mistake the different pathologies [15]. In addition to changes in the hypothalamic-pituitary-gonadal axis, micropenis patients lack the normal coroporal length seen in buried penis [10]. The classification by Maizels et al. is widely referred to, particularly in reference to paediatric buried penis, and identifies buried penis as one of three subgroups of concealed penis [12], along with webbed and trapped. Buried penis is defined as a penis of normal size which is concealed within the pubic tissue due to a lack of fixation of the skin at the base of the penis. By contrast, a trapped penis is secondary to scarring following penile surgery, such as circumcision, and webbed penis is a result of the disappearance of the penoscrotal angle due to abnormally distal extension of scrotal skin over the ventral surface of the penis. Elder clarifies his definition of buried penis (interchangeably used with concealed penis) in children as being caused by an inelasticity of the dartos fascia in infancy and by abundant fat on the abdominal wall in older children [13]. Oh et al. further distinguish between the concealed and buried penis, stating that the aetiology of concealed penis lies in a deficiency of penile skin or inelasticity of the dartos fascia [14]. Buried penis by contrast is secondary to poor fixation of penile skin at the penile base or excessive suprapubic fat [14]. The overarching consensus is thus that childhood buried penis is in the main a congenital condition which can also be seen with postcircumcision scarring.

Ehrlich and Alter suggest that the term buried penis for adults refers to a penile shaft which is buried beneath the surface of the prepubic skin and to a penis which is partially or totally obscured secondary to either obesity or injudicious circumcision [16]. Adult buried penis is viewed largely as an acquired condition with a different pathophysiology from that of children, although some authors consider that some milder forms of dysgenic dartos fasical bands may not be present until adulthood [10], which somewhat blurs the distinction. Warren argues that whereas in boys excess fat is only a contributing factor to penile encroachment, it is causative in men [17]. Male weight gain preferentially involves the abdominal and suprapubic region, and this fat, once present, is difficult to lose through either dieting or exercise. The penile fixity to the pubis results in an apparent length loss as the suprapubic fat pad increases in size [10]. This enveloping fat encourages a moist environment ideal for bacterial growth [9] which results in a cycle of infections which results not only in contracture of the skin surrounding the distal penis, but also in the recruitment of prepubic skin to invaginate the shaft [10], creating a circular scar which traps the penis [9, 18]. Infections are further compounded by the presence of diabetes and its sequelae. Inflammation of surrounding tissue through genital lymphoedema and scarring induced from trauma or circumcisions serves to promote and perpetuate such processes.

There appears to be no reliable data at present about the incidence of buried penis in adults, and it is likely that the number of patients with this condition is far greater than the population presenting to the hospital. No specific BMI value is linked to the probability of having a buried penis [19]. With obesity becoming increasingly prevalent across the world, this is a condition that will be inevitably more frequently present for treatment. Certainly, symptoms of uncontrolled direction of micturition stream, severe sexual dysfunction with painful erections and inability to achieve vaginal penetration, in addition to inability to maintain even basic hygiene or visualize one's penis, will likely also result in complex psychological comorbidities. Surgical intervention however must be embarked on with caution: it is established that obese patients have a high risk of complications [20], particularly in the presence of diabetes, with wound breakdown, infection and systemic postoperative complications. The role of preoperative counseling to address the psychological consequences of this condition and to prepare patients for the postoperative interventions is tremendous and should not be overlooked.

Treatment for buried penis should aim to restore an aesthetic and functional penis [21]. The wide variety of approaches to correcting this problem reflects the different perceptions of aetiology. Having reviewed our results and methods, we retrospectively adapted established treatment algorithms [5] to create a single common pathway for buried penis in children and adults (Figure 1). Through comparison with current literature, each stage can be seen to follow a logical understanding of the underlying pathologies in buried penis. Dissection of the dartos and Buck's fascia with division of chordee is commonly performed, though the approach of the dissection does vary, with some clinicians preferring to make incisions at the penopubic or penoscrotal junction with dissection distally to free the shaft [2–4, 8], some working proximally [10, 22] and others using a combination [5]. In our experience, release from distal to proximal enables clear and safe visualization of the dissection plane and of the neurovascular structures, adhesions, and chordee. Some clinicians induce artificial erections with saline to determine the adequacy of release of adhesions [9, 10], but we have not adopted this into our practice.

Borsellino maintains that the key to correction is release of the abnormal dartos attachments and fixation of the penile skin to Buck's fascia [5]. Reinforcement at the penoscrotal and penopubic angles is widely practiced, though the approach (via stab incisions [3] or dissection), number of sutures (from 2 to 4) [3, 6, 10], and placement of sutures (90 degrees [6], 120 degrees [3], and 180 degrees apart [7, 10]) vary between clinicians. We find the placement of three sutures at 120 degree angles sufficient for penile support and positioning.

The excision of excess fat is largely reserved for adult patients. Whilst liposuction [7] and pubic lipectomy [4] have been described in the treatment of paediatric buried penis, we feel that fat removal in children is largely unnecessary because at a young age, obesity can be self-corrected [6] with judicious exercise and dietary advice. Joseph argues that excision of suprapubic fat in children does not give satisfactory results because the abnormal position of the corporal bodies remains [8], whereas others simply assert that removal is unnecessary and can cause an unsightly ledge in children [5]. Understanding that excess suprapubic and abdominal fat is a significant causative and perpetuating agent in adult buried penis, removing at least some fat is key to a successful outcome. Practice varies from liposuction—acknowledged to be relatively ineffective alone [3, 23, 24]—to excisional mons lipectomy [17], suprapubic lipectomy [22], panniculectomy [18, 21], and abdominoplasty [23, 24] through a host of different approaches. Closure too ranges from anchoring rectus fascia to pubic periosteum [21, 22], to the suspensory ligament [17], through suspension of the superficial base of penis fascia to the deep abdominal fascia [9]. We have found that following a suprapubic lipectomy simple layered closure addresses the fat immediately overlying the dorsum of the penis, permits a significant weight of tissue to be removed, and enables tension-free closure of skin to reduce the risk of wound breakdown. Similar to other clinicians [23], the use of a “W” incision importantly avoids a central line of tension in the abdominal wound.

Finally, penile coverage has been achieved through different combinations and permutations. If no penile shaft skin is identified as being abnormal, direct closure may be possible. In our series, the only patient suitable for direct closure encountered wound breakdown, suggesting that penile skin in affected individuals may be unhealthy even if they appear normal on a macroscopic level. Z-plasties may be used [6, 25], particularly for correction of penoscrotal webs, as may the recruitment of local tissue and flaps [2, 26]. Skin grafting is increasingly favoured in spite of concerns regarding contracture and complications [5, 8]. There is no consensus as to whether outcomes are improved with split thickness skin grafting [2, 9, 10, 22, 26, 27] or full thickness skin grafts [17, 18], or whether they should be applied in a spiral [10] or nonspiral fashion to aid graft take. We apply full thickness grafts to small defects, particularly in children, in a direct nonspiral manner over the ventral surface of the penis and have not encountered any loss of graft, and no functional restriction has been reported by our patients. Our use of hand-fenestrated split thickness sheet graft for larger areas has healed well and aesthetically with an anatomical recreation of the midline raphe. Hand fenestration is not always necessary as multiple quilting sutures forming part of the internal splint allow for fluid drainage. The use of proflavine wool tie-overs, fibrin glue [22], negative pressure systems [28–30], and foam [18] suggests that a dressing which exerts pressure on the graft or replaced skin is helpful. Our experience of proflavine-soaked wool tie-overs in grafts all over the body is strongly positive and is acceptable to patients in the postoperative period. The catheter allows for better aftercare in the postoperative period, with Co-amoxiclav as our preferred antibiotic cover.

## 5. Conclusion

Buried penis is a condition which is difficult to treat both in children and in adults. The classification of buried penis is confusing because the same term is applied to a congenital condition affecting children because of dysgenic fibrous bands as to an acquired condition in adults rooted in obesity. A spectrum exists however linking these poles with circumcision, a causative factor in both adults and children, and the possibility that mild congenital deformities may not present until adulthood when other factors, such as obesity, trauma, or infection, might occur and compound the condition. With the rising prospect of a more obese patient population, plastic, paediatric, and urological surgeons are likely to encounter this uncommon condition more often. With no consensus held over when a buried penis should be corrected in childhood and with no universally accepted paradigm for the surgical management of adults, further work is required to develop our understanding of this condition which carries significant physical and psychological morbidity. We present a modified treatment algorithm to unify and streamline the practice in both adults and children.

Early recognition of buried penis is certainly the key to prompt treatment, as is the local and regional awareness of reconstructive service provision. These patients are often left to lose their desired weight to see the effect of skin shrinkage and the delivery of safe anaesthesia, which may result in patients waiting for years for treatment, so compounding their existing complaints. It is very likely that units offering reconstructive services may have to treat such patients who are still morbidly obese if anaesthetically fit in order to resolve their significant issues regarding function and form.

## Figures and Tables

**Table 1:** Different presentations of buried penis in children and adults.

Presentation	Children (*n* = 4)	Adults (*n* = 5)
Age (years)	6 (8 m–12 y)	51 (28–76)
BMI	Normal	45 (30–48)
Diabetes	—	4
Urinary difficulties	4	4
Sexual dysfunction	—	3
Aesthetic concern	4	3
Recurrent infections	—	4
Fournier's gangrene	—	1
Previous circumcision	—	4
Phimosis	1	1
Hypospadias	1	—

**Table 2:** Complications following buried penis procedures.

Complications	Children	Adults
Infection	0	2
Pain	1	0
Wound dehiscence	0	1
Readmission	0	3
Return to theatre	0	1
Skin loss	1	0

**Table 3:** Postoperative outcomes following buried penis surgery.

On review	Children	Adults
Ongoing urinary problems	0	0
Recurrence of infection	0	0
Improved erectile function	1	3
Effective vaginal penetration	—	1
Altered shaft sensation	1	3
Aesthetic concerns addressed	4	5
Overall satisfaction	All happy	All happy
